# Restoring structural parameters of lipid mixtures from small-angle X-ray scattering data

**DOI:** 10.1107/S1600576720015368

**Published:** 2021-02-01

**Authors:** Petr V. Konarev, Andrey Yu. Gruzinov, Haydyn D. T. Mertens, Dmitri I. Svergun

**Affiliations:** a A. V. Shubnikov Institute of Crystallography, Federal Scientific Research Centre ‘Crystallography and Photonics’ of Russian Academy of Sciences, Leninsky prospekt 59, Moscow, 119333, Russian Federation; b Hamburg Outstation, European Molecular Biology Laboratory, Notkestrasse 85, Hamburg, 22607, Germany

**Keywords:** small-angle X-ray scattering, SAXS, lipids, multi-lamellar vesicles, single unilamellar vesicles, electron density profile

## Abstract

Small-angle X-ray scattering (SAXS) is widely utilized to study soluble macromolecules, including those embedded into lipid carriers and delivery systems such as surfactant micelles, phospho­lipid vesicles and bilayered nanodiscs. To adequately describe the scattering from such systems, one needs to account for both the form factor (overall structure) and long-range-order Bragg reflections emerging from the organization of bilayers, which is a non-trivial task. Presently existing methods separate the analysis of lipid mixtures into distinct procedures using form-factor fitting and the fitting of the Bragg peak regions. This article describes a general approach for the computation and analysis of SAXS data from lipid mixtures over the entire angular range of an experiment. The approach allows one to restore the electron density of a lipid bilayer and simultaneously recover the corresponding size distribution and multilamellar organization of the vesicles. The method is implemented in a computer program, *LIPMIX*, and its performance is demonstrated on an aqueous solution of layered lipid vesicles undergoing an extrusion process. The approach is expected to be useful for the analysis of various types of lipid-based systems, *e.g.* for the characterization of interactions between target drug molecules and potential carrier/delivery systems.

## Abstract 2

An approach to restore the structural parameters of lipid mixtures from small-angle X-ray scattering data is developed.

## 1. Introduction   

**Figure 1:**
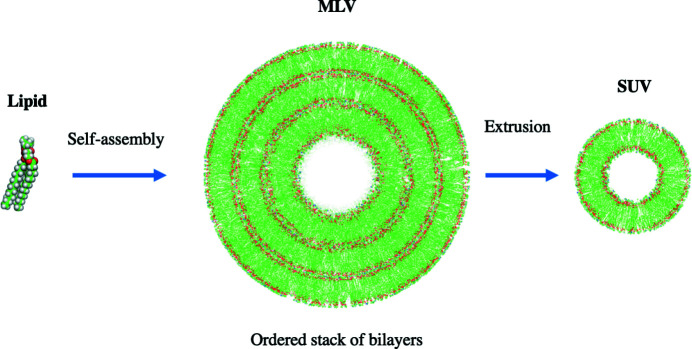
Self-assembly of phospho­lipids into vesicles containing SUVs and MLVs.

Phospho­lipids play an important role in the formation of intracellular and extracellular compartments. They are amphiphilic molecules and self-assemble in aqueous solutions into layered aggregate structures. *In vivo*, phospho­lipids form bilayered components of biological membranes, serving as a scaffold for both hydro­philic and hydro­phobic regions of macromolecules and the separation of cellular components. *In vitro*, phospho­lipids form ordered aggregates such as vesicles and liposomes that adopt single- or multi-lamellar microstructures. Commonly studied bilayered lipid particles formed in aqueous solution include single unilamellar vesicles (SUVs) and multi-lamellar vesicles (MLVs) (Fig. 1 ▸).

Vesicular and lipid systems are important targets in studies of phase transitions and drug delivery mechanisms, and they are also often used to facilitate protein crystallization (Yamashita *et al.*, 2002 ▸; Landau & Rosenbusch, 1996 ▸; Cherezov, 2011 ▸).

Drug delivery systems based on phospho­lipids such as liposomes are widely used for target drug delivery. Liposomes are predominantly used as carriers for hydro­philic molecules that are encapsulated within the aqueous inner volume which is confined by the lipid bilayer. Hydro­phobic drugs can be incorporated into the hydro­phobic part (lipid bilayer) (Bourgaux & Couvreur, 2014 ▸; Malam *et al.*, 2009 ▸). Analysis of the electron density distribution and morphology of the lipid bilayers can give insights into distribution and incorporation of target drugs into liposomes (Schilt *et al.*, 2016 ▸). This approach was shown to be useful for analysing phospho­lipid nanocarriers (Zemlyanaya *et al.*, 2016 ▸; Kiselev *et al.*, 2015 ▸).

Lipid systems are routinely utilized as carriers for membrane proteins to make the latter soluble and stable in aqueous environments, facilitating structural studies. It is therefore important to develop methods for the quantitative analysis of liposome morphology and structure, and to provide tools dedicated to the analysis of polydisperse solutions of lipid vesicles and bilayered nanoparticles such as nanodiscs (Josts *et al.*, 2018 ▸; Flayhan *et al.*, 2018 ▸).

Small-angle X-ray scattering (SAXS) is one of the major methods to study macromolecular solutions, including vesicular systems. Software packages for the analysis of SAXS data from solutions of particles are available, with programs targeted at the biological community (Franke *et al.*, 2017 ▸; Schroer & Svergun, 2018 ▸) and the soft-matter community (Breßler *et al.*, 2015 ▸). As lipid membranes exhibit heterogenous electron density, standard *ab initio* algorithms tailored to the analysis of solutions of homogenous particles (Svergun, 1999 ▸; Franke & Svergun, 2009 ▸; Chacón *et al.*, 1998 ▸) cannot be directly applied. Hybrid approaches that combine *ab initio* modelling with parametrization using simple geometric bodies have been developed, facilitating the construction of models of membrane proteins stabilized by lipid nanodiscs (Skar-Gislinge *et al.*, 2015 ▸; Pérez & Koutsioubas, 2015 ▸).

Various approaches exist to tackle the problem of modelling and fitting small-angle scattering data from model lipid membranes (Pabst *et al.*, 2000 ▸; Pencer & Hallett, 2000 ▸; Pérez & Koutsioubas, 2015 ▸; Skar-Gislinge *et al.*, 2015 ▸; Heftberger *et al.*, 2014 ▸; Zemlyanaya *et al.*, 2016 ▸, 2018 ▸; Konarev *et al.*, 2020 ▸). In most cases, scattering length density (SLD) profiles of symmetric lipid bilayers are represented in the form of step functions (Pencer & Hallett, 2000 ▸) or Gaussians (Pabst *et al.*, 2000 ▸). Improved SLD models for the determination of asymmetric bilayer structure in unilamellar vesicles have also been developed (Brzustowicz & Brunger, 2005 ▸), including detailed models for combining both X-ray and neutron scattering (Kučerka *et al.*, 2007 ▸; Pabst *et al.*, 2010 ▸; Marquardt *et al.*, 2015 ▸).

Lipid bilayers can exhibit significant bending fluctuations of entropic origin and scattering techniques are also employed to probe the membrane elasticity. In MLVs, these effects lead to a power-law decay of the correlation function and characteristic shapes of Bragg peaks (Pabst *et al.*, 2010 ▸). Membrane elasticity is assessed from the shape analysis of the Bragg peaks using the modified Caille theory (Caille, 1972 ▸; Zhang *et al.*, 1994 ▸). The fluctuation degree is determined by the Caille parameter η, which is a function of the bilayer bending modulus and the bulk modulus of inter-bilayer compression. For this analysis, X-ray scattering has an edge over neutron studies because of the higher resolution of SAXS data compared with that typically achieved with neutrons.

Here, we present a program, *LIPMIX*, that builds upon the methodology first described by Pabst *et al.* (2000 ▸) to derive structural parameters of polydisperse multilamellar lipid mixtures (including bilayer electron density, vesicle size distribution and multilamellar organization of the vesicles) utilizing the scattering data collected over the entire angular range of a SAXS experiment. Modern SAXS facilities provide broad angular ranges in a single measurement with excellent resolution, and the approach implemented in *LIPMIX* for the simultaneous analysis of diffraction and diffuse scattering can be readily applied for the analysis of such data. We demonstrate the use of the method to analyze the SAXS data from lipid vesicles undergoing an extrusion process in aqueous solution. Parameters are extracted and a model constructed that describes the transition of the system from MLV to SUV particles and represents the mixture of polydisperse MLV and SUV species.

## 2. Materials and methods   

### 2.1. Sample preparation   

Dimyristoylphosphatidylcholine (DMPC) and dipalmitoylphosphatidylcholine (DPPC) were purchased from Anatrace (Maumee, OH, USA) and used without further purification. Lipids were dissolved in MilliQ grade water incubated for 1 h at 50°C for total hydration, and then the solutions were rapidly frozen in liquid nitro­gen and incubated for 10 min at 50°C. This freeze–thawing cycle was repeated five times to prepare the liposomal suspensions for the extrusion process. Freeze–thawed suspensions were repeatedly extruded at 50°C through polycarbonate membranes (pore sizes: 30, 50, 100 and 200 nm) using a 1 ml mini-extruder (Avanti Polar Lipids). Between each extrusion cycle samples were vortexed and spun down in a centrifuge for 1 min at 1000 r min^−1^ and room temperature. Aliquots of the resulting solutions without any undissolved sediment were transferred to standard Eppendorf tubes and stored at +4°C.

### 2.2. SAXS measurements   

SAXS data were collected at the P12 beamline of the European Molecular Biology Laboratory (EMBL) at the PETRA III storage ring, DESY Hamburg (Blanchet, Spilotros*, et al.*, 2015 ▸). The samples were loaded using a robotic sample changer (Round *et al.*, 2015 ▸) into a flow-through capillary of 1.7 mm diameter. All data sets were collected as 20 consecutive 50 ms frames using a Pilatus 6M pixel detector (DECTRIS, Switzerland). The data collection and reduction were performed using *BECQUEREL* (Hajizadeh *et al.*, 2018 ▸) and the *SASFLOW* pipeline (Franke *et al.*, 2012 ▸), including the comparison of frames for radiation damage, averaging and buffer subtraction. The sample-to-detector distance was 3.1 m; the X-ray wavelength λ was 1.24 Å. The averaged frames were normalized to the transmitted beam using a beamstop with an integrated photodiode (Blanchet, Hermes *et al.*, 2015 ▸). The temperature was kept constant at 30°C for DMPC and 45°C for DPPC, *i.e*. above the temperature of the main phase transition of the corresponding lipid (24 and 41°C for DMPC and DPPC, respectively). A summary of the experimental data collection is given in Table 1 ▸. An independent experimental session on the temperature dependence of unextruded and extruded DPPC vesicles was carried out in the range from 10 to 40°C.

### 2.3. Scattering from polydisperse multilamellar lipid mixtures   

In the derivations below, we utilize the fact that the SAXS data collected from symmetric lipid vesicles can be well approximated by the product of the form factor of a thin spherical shell *F*_TS_ (defining the vesicle size) and the form factor of a flat lipid bilayer *F*_FB_ (containing information about the electron density across the bilayer). This ‘separated form factor’ (SFF) approximation is valid when the vesicle size is much larger than the bilayer thickness (Pencer *et al.*, 2006 ▸; Kiselev *et al.*, 2002 ▸). The ordered behaviour of multiple bilayers inside the vesicle can be taken into account by an additional interbilayer structure factor multiplier term (Heftberger *et al.*, 2014 ▸).

The intensity from a dilute polydisperse mixture of MLVs can be represented as follows:
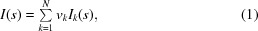
where *s* = 4πsinθ/λ, 2θ is the scattering angle, *N* is the number of MLV particles with different bilayer structures, and *ν_k_* and *I_k_*(*s*) are the corresponding volume fractions and partial scattering intensities from these MLVs. Using the SFF approximation and taking into account vesicle size polydispersity and variability of multilamellar organization, each partial intensity can be expressed as
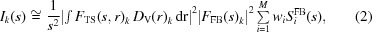
where *D*_V_(*r*)*_k_* is the volume size distribution of vesicles, *F*_TS_(*s*, *r*)*_k_* is the form factor of a thin spherical shell with radius *r*, *F*_FB_(*s*)*_k_* is the form factor of the flat lipid bilayer of the *k*th component in the mixture, *M* is the total number of MLV particles with different multilamellar organization, 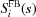 is the interbilayer structure factor of evenly spaced flat bilayers and *w_i_* is the occupancy factor for MLV particles with a given number of ordered lipid bilayers. In the calculations below, the volume distribution of MLVs *D*_V_(*r*) is parametrized by a monomodal Schulz distribution with a mean radius *R* and width σ (Schulz, 1935 ▸).

The form factor *F*_FB_(*s*) is given by the Fourier transform of the electron density profile of the bilayer. This can be approximated by five Gaussian functions [similar to the approach taken by Pabst *et al.* (2003 ▸)]: 
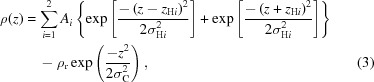
where the first four Gaussian terms of width σ_H*i*_ centred at *z*_H*i*_ (*i* = 1, 2) represent the hydro­philic phospho­lipid polar headgroups to model both symmetric and asymmetric density profiles. The fifth Gaussian term of width σ_C_ at the centre of the bilayer shell accounts for the hydro­phobic hydro­carbon chains and ρ_r_ is the ratio of the electron density of the hydro­carbon chains to that of the headgroups.

The interbilayer structure factor *S* ^FB^(*s*) from *L* evenly spaced flat bilayers of finite size resulting in the appearance of Bragg peaks is calculated according to the modified Caille theory (Caille, 1972 ▸; Zhang *et al.*, 1994 ▸):
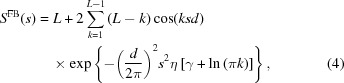
where *L* is the total number of ordered flat bilayers in the vesicle, *d* is the lamellar repeat distance and η is the Caille parameter, which is a measure for the bilayer bending fluctuations; γ is the Euler constant.

### 2.4. Restoration of the structural parameters of the mixtures   

Equations (1)–(4) [Disp-formula fd3] [Disp-formula fd4] allow one to parameterize the scattering from a polydisperse mixture of MLV particles in terms of a few structural parameters (Table 2 ▸). The program *LIPMIX* optimizes these parameters to fit the given experimental data using a quasi-Newton minimization procedure developed by Broyden–Fletcher–Goldfarb–Shanno (Gill *et al.*, 1981 ▸). The parameters of the initial approximation are estimated from the experimental data and simple bounds are imposed on the variables, reflecting physically justified limitations of the system. Thus, the initial average vesicle radius *R_k_* is obtained from the radius of gyration *R*_g_ calculated from the Guinier approximation (Guinier, 1939 ▸), while the vesicle size polydispersity can be varied in a broad interval [0.02*R*_g_–0.5*R*_g_]. For the electron density parameters of the lipid bilayer, the centres of phospho­lipid polar headgroups *z*_H*i*_ can be varied within the interval [1.5–2.5] nm, and their widths are allowed to change within [0.1–0.3] nm, thus providing an overall bilayer thickness of 4.5–6.5 nm. *LIPMIX* can be run automatically using a command file with model specifications and initial values of the parameters as well as their upper and lower limits. Python scripts can be used to generate command files with the desired number of combinations of MLV components and run them locally or in parallel on a computer cluster in order to rapidly screen the multilamellar organization of the system (available at https://git.embl.de/agruzinov/lipmix_utils).

## 3. Results and discussion   

### 3.1. Simulated example   

**Figure 2:**
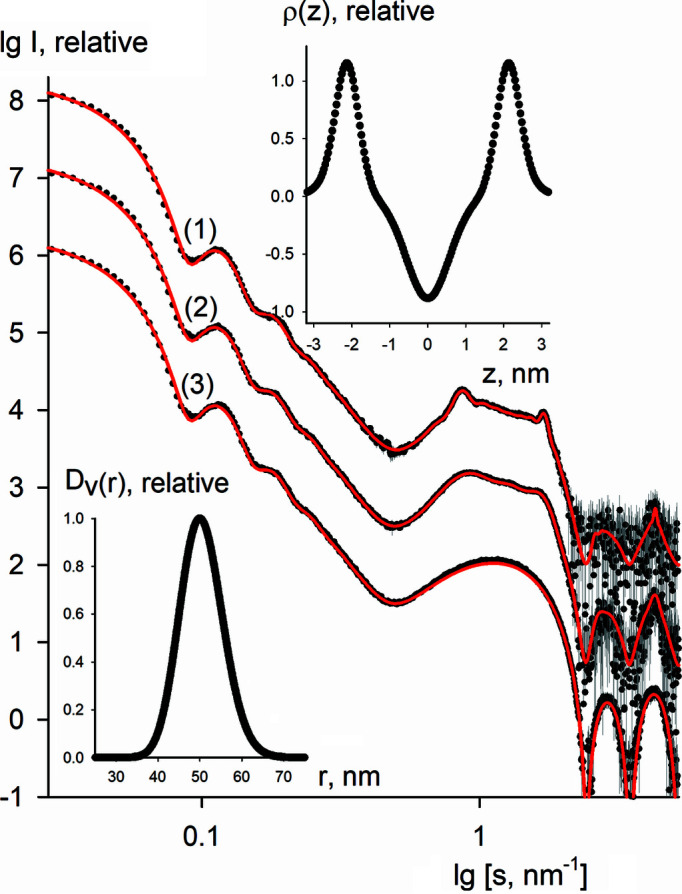
Simulated scattering curves from the lipid mixtures (dots with error bars) and the fits obtained by *LIPMIX* (red solid curves). Curve 1 corresponds to a mixture of SUVs and MLVs (with up to seven ordered layers), curve 2 to a mixture of SUVs and MLVs (with up to three ordered layers), and curve 3 to SUV particles. The vesicle size distribution *D*_V_(*r*) and the electron density profile of the lipid bilayer ρ(*z*) are shown in the insets.

We have first used a simulated data set to test the performance and reliability of the algorithm. For this, we selected three cases of lipid mixtures: (i) only SUV particles, (ii) SUVs and MLVs with up to three ordered layers, and (iii) SUVs and MLVs with up to seven ordered layers. The average radius of the particles was equal to 50 nm, the polydispersity degree was 10%, the electron density had a symmetric profile, the bilayer thickness was 6.5 nm and the Caille parameter was 0.10 for the MLVs. A relative Poisson error of 5% was added to the simulated data (displayed in Fig. 2 ▸ as dots with error bars); the corresponding vesicle size distribution and electron density bilayer profile are shown in the insets of Fig. 2 ▸.

Different starting approximations (with relative deviations of up to 40% from the true parameter values) were tested and in all cases the algorithm converged to the true solution. The fits to the simulated data sets obtained by *LIPMIX* are shown in Fig. 2 ▸ as red solid lines. The obtained results demonstrate that the program reliably restores the structural parameters of lipid mixtures.

### 3.2. Analysis of fully formed vesicles after extrusion process   

**Figure 3:**
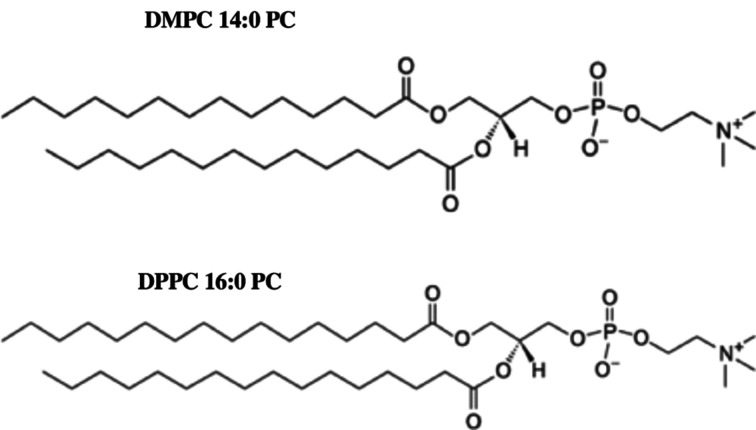
Chemical structures of 1,2-dimyristoyl-*sn*-glycero-3-phospho­choline and of 1,2-dipalmitoyl-*sn*-glycero-3-phospho­choline.

**Figure 4:**
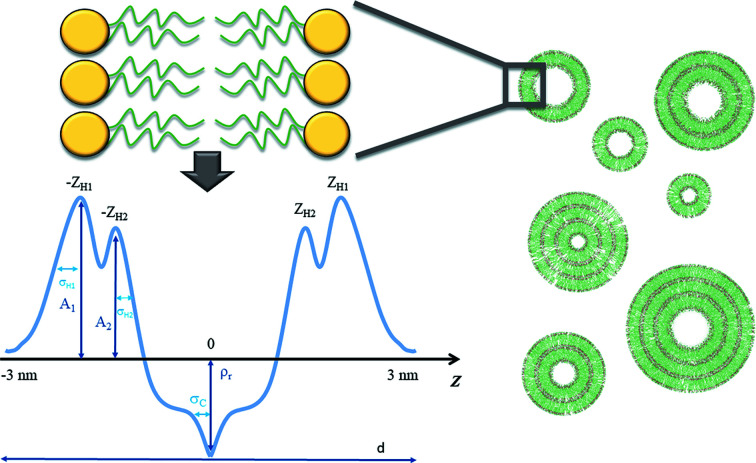
Model parameters of the lipid bilayer that are used for representation of the electron density of the bilayer as a sum of Gaussian functions (left panel). The positive electron density with asymmetric peaks represents the polar heads of the lipids. The central negative peak of the density profile corresponds to the hydro­phobic tails. The lamellar repeat distance (*d*) is shown at the bottom. In the right panel the organization of the SUV and MLV mixture in solution is shown.

We have further tested the performance of the approach using the SAXS data from evolving DMPC/DPPC vesicles recorded during an extrusion process. The chemical structure of DMPC and DPPC lipids is displayed in Fig. 3 ▸, the organization of the MLV particles is shown schematically in Fig. 4 ▸ and the structure parameters required to describe this system are given in Table 2 ▸.

**Figure 5:**
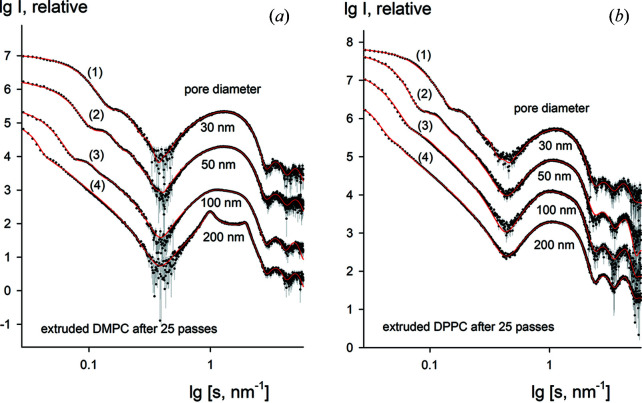
Experimental scattering patterns (dots with error bars) of the extruded vesicles after 25 passes through the extruder for DMPC (*a*) and DPPC (*b*) using different pore diameters (curve 1 – 30 nm, curve 2 – 50 nm, curve 3 – 100 nm, curve 4 – 200 nm) and the fits obtained by *LIPMIX* (red solid lines).

Fig. 5 ▸ (dots with error bars) presents the SAXS data collected from fully extruded vesicles obtained after 25 passes through polycarbonate membranes. Such a repeating procedure is expected to yield homogeneous vesicle populations with no significant size variations of the vesicles. Polycarbonate membrane filters of different pore sizes (30, 50, 100 and 200 nm) were used. After running *LIPMIX*, good quality fits to the experimental data are obtained for all filter sizes over the entire measured angular range (Fig. 5 ▸, solid lines).

**Figure 6:**
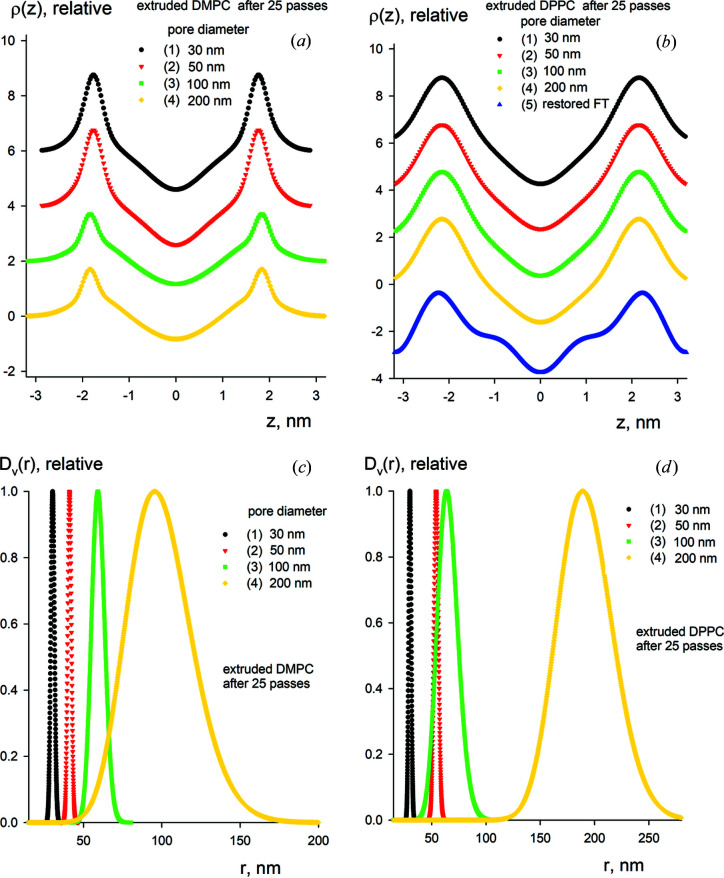
Electron density profiles of lipid bilayers (*a*), (*b*) and vesicle size distributions (*c*), (*d*) for DMPC (left side) and DPPC (right side) after 25 passes through the extruder using different pore diameters. Curve notations (1–4) are the same as in Fig. 5 ▸. The electron density of DPPC obtained using direct computation from the diffraction peaks is denoted as curve 5 (*b*).

The restored structural parameters are summarized in Table 3 ▸, and Fig. 6 ▸ displays the obtained electron density profiles of the lipid bilayers and size distributions of the vesicles for DMPC and DPPC. Notably, the density profiles and size distributions differ significantly for the two types of vesicles, although the bilayer thickness of DMPC is similar to that of DPPC (∼6 nm). For DMPC, the density profile displays narrow peaks for the hydro­philic head group regions of the lipid bilayer. The core region, corresponding to the hydro­phobic hydro­carbon lipid tails, shows a central broad electron density peak. For DPPC, there are broad peaks appearing in the head group regions together with a broad region of electron density observed in the hydro­phobic core. The density profiles appear to be relatively insensitive to the pore diameters used for the extrusion. The restored profiles of the DPPC vesicles in Fig. 6 ▸(*b*) (curves 1–4) are in good agreement with the profile obtained by a direct computation of the form factors from the diffraction peaks using a Fourier reconstruction [Fig. 6 ▸(*b*), curve 5] described elsewhere (Torbet & Wilkins, 1976 ▸; Zhang *et al.*, 1994 ▸) for a polydisperse solution of unextruded multilamellar liposomes from DPPC. The multilamellar organization of lipid vesicles after the extrusion process corresponds to SUV particles.

For the vesicle size distributions, the situation is more complicated. For both DMPC and DPPC particles, a clear correlation is observed between the average vesicle radii and the pore diameters, and the vesicle radii are proportional to the pore diameters. However, on average, the external diameter of the vesicles remains slightly larger than the designed pore diameter, reflecting the ability of the particles to compress/deform to a certain extent while remaining intact as they pass through the membrane pores. Such behaviour has been noted previously and depends on the pressure applied during extrusion (MacDonald *et al.*, 1991 ▸; Frisken *et al.*, 2000 ▸).

The SFF approximation, allowing one to model the bilayer density with any integrable analytical or numeric function (Kiselev *et al.*, 2002 ▸), has a fundamental advantage over the hollow sphere model, but the SFF is valid only when the vesicle size significantly exceeds the bilayer thickness. Comparisons of the scattering by uniform spherical shells with that by shells with a varying ratio of the bilayer thickness to the liposome radius demonstrated that the SFF approximation provides a reasonable agreement with the analytic calculations up to the ratio of 0.5 (Pencer *et al.*, 2006 ▸). Typically, the bilayer thickness is around 6 nm. The SFF approximation is valid in a broad variety of practical cases, and in particular, for the presented examples (where the smallest vesicles have a diameter of 60 nm), the approximation fits the SAXS data.

One should, however, note that the five-Gaussian approximation employed to represent the bilayer density profile in the present algorithm may have limitations, for example when restoring the finer details of the profiles from lipids containing several components (lipid rafts) (Heberle *et al.*, 2016 ▸). In these cases one may consider alternative approaches developed earlier, *e.g.* that of Oliveira *et al.* (2012 ▸), where the density profile is modelled by a set of equally spaced Gaussian functions and the smoothness of the profile is achieved by the Lagrange multiplier parameter during a minimization procedure. On the other hand, the algorithm of Oliveira *et al.* (2012 ▸) does not account for size distributions of the vesicles and assumes the presence of multilamellar vesicles with only a certain number of ordered layers.

### 3.3. Evolution of vesicle parameters during extrusion   

**Figure 7:**
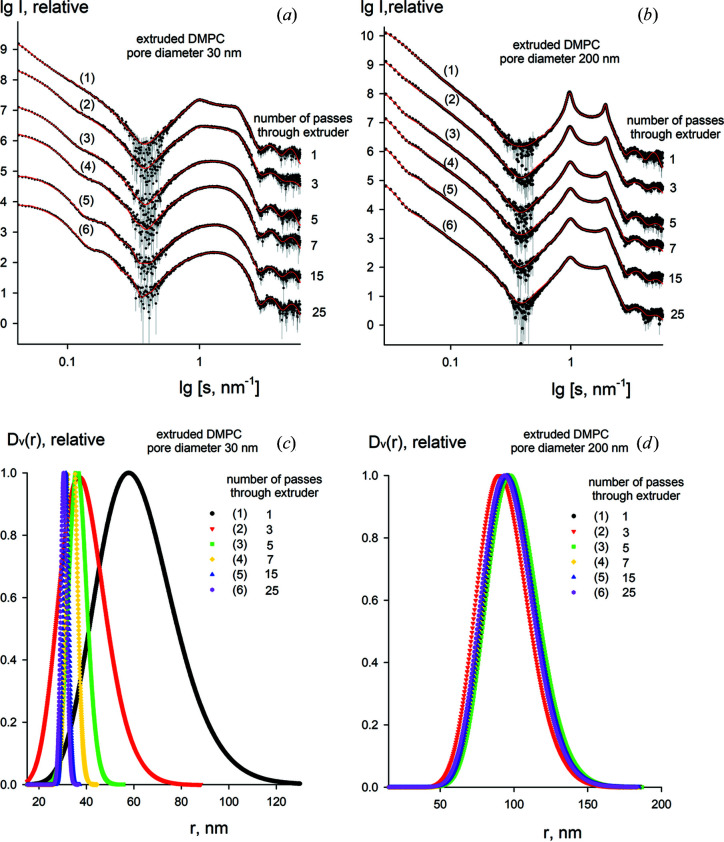
Experimental scattering patterns (*a*), (*b*) and the restored size distributions of the vesicles (*c*), (*d*) obtained by *LIPMIX* for DMPC vesicles during the extrusion process (from 1 to 25 membrane passes) using the pore diameters 30 nm (left side) and 200 nm (right side). Experimental data are shown as dots with error bars and the calculated fits as red solid lines.

To further verify the reliability and versatility of *LIPMIX*, we analysed the evolution of the vesicle structural parameters during the extrusion process. Experimental data were collected from solutions of extruded DMPC particles with varying number of membrane passes (from 1 to 25) using large-pore (200 nm) and small-pore (30 nm) membranes. The SAXS data and computed fits by *LIPMIX* are presented in Figs. 7 ▸(*a*) and 7 ▸(*b*) and the calculated structural parameters are summarized in Table 4 ▸.

The evolution of the structural parameters with extrusion clearly reveals that the number of MLV particles in solution decreases in line with the number of passes through the polycarbonate membranes. When repeated extrusion is performed (20–25 passes) using a 30 nm pore diameter membrane only SUV particles are left in solution (Table 4 ▸). The electron density profiles do not significantly change with the number of passes and are similar to those presented in Fig. 6 ▸(*a*), whereas the vesicle size distributions do depend both on the membrane pore diameter and on the number of passes in the extrusion process [Figs. 7 ▸(*c*) and 7 ▸(*d*)]. Interestingly, for the large pore diameter (200 nm) the average vesicle radius remains constant at around 100 nm with a relatively high degree of polydispersity (around 15–20%). However, for the small pore diameter (30 nm) the average vesicle radius significantly decreases with the number of passes (from 61 to 30 nm) and the polydispersity of the vesicles drops from 25 to 5%, suggesting that SUV particles with reduced polydispersity are formed. These results are in good agreement with previous studies (MacDonald *et al.*, 1991 ▸) and further confirm that the approach of *LIPMIX* is robust and applicable to polydisperse solutions of lipid aggregates.

### 3.4. Temperature dependence of the structural parameters of the DPPC vesicles   

**Figure 8:**
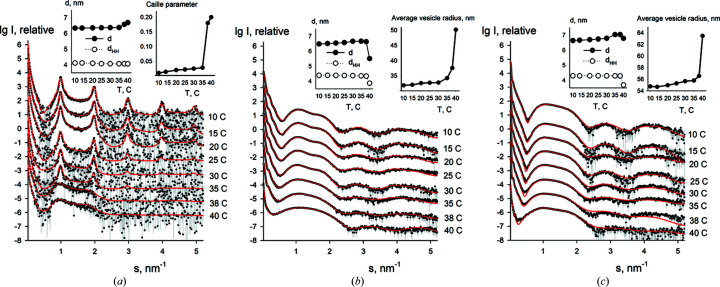
Experimental scattering patterns (dots with error bars) of the unextruded DPPC vesicles (*a*) and extruded DPPC vesicles [(*b*) 30 nm pore diameter, (*c*) 100 nm pore diameter] within the temperature range 10–40°C and the fits obtained by *LIPMIX* (red solid lines). The insets contain the temperature dependence of the lamellar repeat distance (*d*), the head-to-head distance (*d*_HH_), the degree of vesicle bending fluctuation (Caille parameter) (for unextruded DPPC) and the average vesicle radius (for extruded DPPC).

We have also analyzed the dependence of the structural parameters of the unextruded multilamellar DPPC vesicles as well as extruded DPPC vesicles (with 30 and 100 nm pore diameters, respectively) on the temperature within 10–40°C. The SAXS data and the fits computed by *LIPMIX* are presented in Fig. 8 ▸. For unextruded vesicles, the MLVs are present in solution at all studied temperatures: they consist of 10–11 ordered bilayers at 10–35°C, whereas the MLVs with only 2–3 ordered bilayers are left at 38–40°C [Fig. 8 ▸(*a*)]. The lamellar repeat distance (*d*) gradually increases with increasing temperature from 6.3 to 6.7 nm, while the head-to-head distance (*d*_HH_) (the distance between the electron density peaks from phospho­lipid polar headgroups of the bilayer) remains almost constant at around 4.3 nm [Fig. 8 ▸(*a*), left inset]. The average vesicle radius is 110–120 nm and shows a high degree of polydispersity (around 20%). The bilayer bending fluctuations (proportional to the Caille parameter) have also a significant increase at about 38°C [Fig. 8 ▸(*a*), right inset].

For the extruded DPPC vesicles (30 nm pore diameter) MLVs with up to two ordered layers are present at 10–30°C, whereas only SUVs are left at 35–40°C. The lamellar repeat distance (*d*) gradually increases with increasing temperature from 6.4 to 6.6 nm (within 10–38°C) and drops to 5.6 nm at 40°C [Fig. 8 ▸(*b*), left inset] near the phase transition temperature of DPPC (*T*_c_ = 41.3°C). At the same time, the average vesicle radius gradually increases from about 32 nm (at 10°C) to 37.5 nm (at 38°C) and displays a jump to about 50 nm at 40°C [Fig. 8 ▸(*b*), right inset]. The polydispersity is relatively low at 10–35°C (about 5%) and increases at 38–40°C (to 15%).

The extruded DPPC vesicles with 100 nm pore diameter form only SUVs at all studied temperatures. The lamellar repeat distance (*d*) increases monotonically from 6.6 to 7.0 nm (within 10–38°C) and slightly decreases to 6.8 nm at 40°C [Fig. 8 ▸(*c*), right inset]. At the same time, the average vesicle radius gradually increases from 54.7 nm (at 10°C) to 56.5 nm (at 38°C) and jumps to 63.5 nm at 40°C [Fig. 8 ▸(*c*), right inset]. The polydispersity is relatively low (about 3–4%) at 10–38°C and increases to 25% at 40°C. The obtained results are in line with the expected behaviour of the DPPC systems (Pabst *et al.*, 2004 ▸; Soloviov *et al.*, 2012 ▸) and further confirm the usefulness of *LIPMIX* for the studies of polydisperse multilamellar vesicles.

## 4. Conclusions   

A new approach was developed to evaluate the structural parameters of polydisperse lipid mixtures containing multilayered particles from solution SAXS data. The approach allows one to restore the overall vesicle size and polydispersity as well as the multilamellar organization of lipid species and electron density profiles of the lipid bilayers. The algorithm, implemented in the computer program *LIPMIX*, was tested on both simulated and experimental data from DPPC and DMPC vesicles at different stages of the membrane extrusion process. The reconstructed electron density profiles of the vesicles are in good agreement with the results obtained from direct Fourier reconstructions (note that the latter approach may not be applicable at low and medium sample concentrations, where the higher-order diffraction peaks become too noisy or simply not visible).

The test applications of *LIPMIX* to the DPPC and DMPC systems indicate that before extrusion the DMPC/DPPC lipids form MLVs consisting of 20–25 lipid bilayers. During the extrusion process the number of bilayers decreases, and after 10–15 passes the vesicles contain only 4–5 bilayers. After 20–25 passes only SUV particles are left in solution, except for the DMPC particles going through the filter of large pore diameter (200 nm) where the multilamellar organization is preserved. The vesicle size distributions depend strongly on the membrane pore diameter and on the number of membrane passes during the extrusion process.

The results obtained demonstrate that *LIPMIX* allows one to directly analyse structural polydispersity and composition of phospho­lipid systems. In particular, the approach can be applied to lipid vesicles undergoing an extrusion process and/or a phase transition. *LIPMIX* is included in the *ATSAS* software (as of release 3.0; Manalastas-Cantos *et al.*, 2021 ▸), freely available for academic users at https://www.embl-hamburg.de/biosaxs/software.html.

## Figures and Tables

**Table:** (*a*) Sample details.

	1,2-Dimyristoyl-*sn*-glycero-3-phospho­choline (DMPC)	1,2-Dihexa­decanoyl-*sn*-glycero-3-phospho­choline (DPPC)
Source	Anatrace (Maumee, OH, USA): Product ID: D514	Anatrace (Maumee, OH, USA): Product ID: D516
Molecular mass *M* from chemical composition (monomer) (Da)	677.9	734.039
Solvent composition	Deionized water (MilliQ grade)	

**Table:** (*b*) SAS data collection parameters.

Instrument/data processing	EMBL P12 (PETRA III, DESY, Hamburg)
Wavelength (Å)	1.24
Beam geometry (size, sample-to-detector distance)	0.15 × 0.25 mm^2^, 3.1 m
*s* measurement range (Å^−1^)	0.003–0.700
Absolute scaling method	None
Basis for normalization to constant counts	To transmitted intensity by beamstop counter
Method for monitoring radiation damage	Frame comparison
Exposure time, number of exposures	1 s (20 × 0.05 s)
Sample temperature (°C)	30–45

**Table:** (*c*) Software employed for SAS data reduction, analysis and interpretation.

SAS data reduction	*I*(*s*) versus *s* using *RADAVER* (*ATSAS 2.8.3*; Franke *et al.*, 2017 ▸) and solvent subtraction using the *SASFLOW* pipeline (Franke *et al.*, 2012 ▸)
Basic analyses: computation of theoretical scattering, size distribution, electron density profile	*LIPMIX*

**Table:** (*d*) SASBDB IDs for data and models.

DMPC (extrusion series 25 passes)				DPPC (extrusion series 25 passes)			
Membrane filter (nm)				Membrane filter (nm)			
30	50	100	200	30	50	100	200
SASDG52	SASDG62	SASDG72	SASDG82	SASDG92	SASDGA	SASDGB2	SASDGC2

**Table 2:** Structural parameters for the lipid mixtures containing a number of vesicles with different electron density bilayer profiles The *k*th individual component in a lipid mixture (*k* = *1*–*N*).

Volume fraction {*v_k_*}	Average vesicle radius and size polydispersity {*R_k_*, σ*_k_*}	Electron density profile {*A_i_*, *z*_H*i*_, σ_H*i*_, ρ_r_, σ_c_} (*i *= 1, 2)	Multilamellar organization
			SUV {*w_i_*, *L_i_*}, *L_i_* = 1
			MLV {*w_i_*, *L_i_*}, *L_i_* ≥ 2 (*i* = 1–*M*)
			Caille parameter η

**Table 3:** The structure of extruded DMPC/DPPC vesicles The optimized parameters describing the lipid bilayer structure, the overall sizes of MLVs and their multilamellar organization are obtained by *LIPMIX* from the experimental SAXS data of DMPC and DPPC particles in aqueous solutions after 25 passes through the extruder. The error estimates for the model parameters were obtained as a standard deviation of optimized parameters from successful *LIPMIX* reconstructions.

	DMPC				DPPC			
Membrane pore diameter	30 nm	50 nm	100 nm	200 nm	30 nm	50 nm	100 nm	200 nm
Parameters of the lipid bilayer								
*z*_H1_ (nm)	1.86 ± 0.02	1.83 ± 0.02	1.82 ± 0.02	1.84 ± 0.02	2.14 ± 0.03	2.13 ± 0.03	2.12 ± 0.03	2.15 ± 0.03
σ_H1_ (nm)	0.27 ± 0.01	0.26 ± 0.01	0.25 ± 0.01	0.27 ± 0.01	0.32 ± 0.01	0.33 ± 0.01	0.34 ± 0.01	0.32 ± 0.01
*A*_2_/*A*_1_	1.78 ± 0.04	1.78 ± 0.04	1.76 ± 0.04	1.74 ± 0.04	1.78 ± 0.04	1.74 ± 0.04	1.75 ± 0.04	1.76 ± 0.04
*z*_H2_ (nm)	1.71 ± 0.01	1.76 ± 0.01	1.80 ± 0.01	1.78 ± 0.01	2.16 ± 0.03	2.15 ± 0.03	2.14 ± 0.03	2.17 ± 0.03
σ_H2_ (nm)	0.13 ± 0.01	0.12 ± 0.01	0.12 ± 0.01	0.14 ± 0.01	0.34 ± 0.02	0.33 ± 0.02	0.32 ± 0.02	0.35 ± 0.02
ρ_r_	1.53 ± 0.02	1.50 ± 0.02	1.54 ± 0.02	1.53 ± 0.02	1.74 ± 0.02	1.63 ± 0.02	1.65 ± 0.02	1.61 ± 0.02
σ_C_ (nm)	0.42 ± 0.03	0.40 ± 0.03	0.45 ± 0.03	0.43 ± 0.03	0.42 ± 0.03	0.45 ± 0.03	0.44 ± 0.03	0.43 ± 0.03

Parameters of the MLV size distribution								
*R* (nm)	30 ± 1	41 ± 1	59 ± 1	100 ± 2	30 ± 1	54 ± 1	65 ± 1	192 ± 3
σ (nm)	1.2 ± 0.1	1.3 ± 0.1	4.2 ± 0.2	21 ± 1	1.2 ± 0.1	2.1 ± 0.2	9.4 ± 0.5	25 ± 1

Structural organization								
Caille parameter η	–	–	–	0.10 ± 0.01	–	–	–	–
Maximum number of ordered bilayers (SUV/MLV)	SUV	SUV	SUV	SUV + MLV (up to 8 layers)	SUV	SUV	SUV	SUV

**Table 4:** The evolution of DMPC vesicles during the extrusion process The optimized parameters describing the lipid bilayer structure, the overall sizes of MLVs and their multilamellar organization are obtained by *LIPMIX* from the experimental SAXS data of DMPC particles in aqueous solutions with different numbers of passes through the extruder. The error estimates for the model parameters were obtained as a standard deviation of optimized parameters from successful *LIPMIX* reconstructions.

	Membrane pore diameter (30 nm)				Membrane pore diameter (200 nm)			
Number of passes	1	5	9	25	1	5	9	25
Parameters of the lipid bilayer								
*z*_H1_ (nm)	1.89 ± 0.02	1.92 ± 0.02	1.90 ± 0.02	1.86 ± 0.02	1.85 ± 0.02	1.82 ± 0.02	1.82 ± 0.02	1.84 ± 0.02
σ_H1_ (nm)	0.28 ± 0.01	0.27 ± 0.01	0.26 ± 0.01	0.27 ± 0.01	0.29 ± 0.01	0.28 ± 0.01	0.25 ± 0.01	0.27 ± 0.01
*A*_2_/*A*_1_	1.81 ± 0.04	1.82 ± 0.04	1.86 ± 0.04	1.78 ± 0.04	1.73 ± 0.04	1.71 ± 0.04	1.75 ± 0.04	1.74 ± 0.04
*z*_H2_ (nm)	1.65 ± 0.01	1.64 ± 0.01	1.65 ± 0.01	1.71 ± 0.01	1.79 ± 0.01	1.80 ± 0.01	1.75 ± 0.01	1.78 ± 0.01
σ_H2_ (nm)	0.17 ± 0.01	0.14 ± 0.01	0.15 ± 0.01	0.13 ± 0.01	0.17 ± 0.01	0.16 ± 0.01	0.15 ± 0.01	0.14 ± 0.01
ρ_r_	1.58 ± 0.02	1.65 ± 0.02	1.64 ± 0.02	1.53 ± 0.02	1.56 ± 0.02	1.56 ± 0.02	1.55 ± 0.02	1.53 ± 0.02
σ_C_ (nm)	0.41 ± 0.03	0.44 ± 0.03	0.45 ± 0.03	0.42 ± 0.03	0.42 ± 0.03	0.40 ± 0.03	0.41 ± 0.03	0.43 ± 0.03

Parameters of the MLV size distribution								
*R* (nm)	61 ± 1	37 ± 1	33 ± 1	30 ± 1	96 ± 2	100 ± 2	99 ± 2	100 ± 2
σ (nm)	14 ± 1	3.5 ± 0.2	1.2 ± 0.1	1.2 ± 0.1	14 ± 1	17 ± 1	17 ± 1	21 ± 1

Structural organization								
Caille parameter η	–	–	–	–	0.11 ± 0.01	0.12 ± 0.01	0.09 ± 0.01	0.10 ± 0.01
Maximum number of ordered bilayers (SUV/MLV)	SUV	SUV	SUV	SUV	SUV + MLV (up to 8 layers)	SUV + MLV (up to 8 layers)	SUV + MLV (up to 8 layers)	SUV + MLV (up to 8 layers)
